# Serendipitous diagnosis of aortic coarctation by bilateral parvus et tardus renal Doppler flow pattern

**DOI:** 10.1186/1476-7120-5-44

**Published:** 2007-11-26

**Authors:** Mohammad Kazem Tarzamni, Nariman Nezami, Mohammad Reza Ardalan, Jalal Etemadi, Hamid Noshad, Fatemeh Gatreh Samani, Mehrnoush Toufan

**Affiliations:** 1 Department of Radiology, Tabriz University of Medical Sciences, Tabriz, Iran; 2 Drug Applied Research Center, Tabriz University of Medical Sciences, Tabriz, Iran; 3 Young Researchers Club, Tabriz, Iran; 4 Department of Nephrology, Tabriz University of Medical Sciences, Tabriz, Iran; 5 Department of Cardiology, Tabriz University of Medical Sciences, Tabriz, Iran

## Abstract

**Background:**

Aorta Coarctation (AC) is uncommon condition that in most adult patients is asymptomatic. Diagnosis of AC is made during routine physical examination by detection of Blood Pressure (BP) difference between arm and leg.

**Aim:**

To describe a novel renal artery Doppler flow pattern pathognomonic of aortic coarctation.

**Methods:**

We enrolled 4 consecutive patients referred to renal artery Doppler Ultrasonography (DU) for diagnostic work-up of secondary arterial hypertension. All met the following inclusion criteria: 1) arterial hypertension at age <30 years; 2) referred for renal DU to rule out renovascular hypertension.

**Results:**

We found in all 4 patients (age range 10 to 27 years) a bilateral "parvus-tardus" renal Doppler flow pattern. In all, echocardiographic and angiographic work-ups showed aortic coarctation.

**Conclusion:**

Careful physical examination should be performed in all hypertensive patients. Furthermore, the suspicion of AC can be raised by a bilateral renal arteries "parvus-tardus" Doppler flow pattern in young hypertensive patients screened for secondary hypertension.

## Background

Aortic Coarctation (AC) is a congenital malformation of the aorta accounting for 6–8% of congenital cardiovascular disease [1]. This disease should be diagnosed and treated early in life, because untreated AC develops systemic hypertension and subsequent morbidity and mortality due to cardiovascular diseases [2]. Although suspicion of AC raises during routine physical examination by detecting Blood Pressure (BP) difference between arm and leg [1], AC can be presented during adulthood with isolated or resistant hypertension and without significant difference in BP between the upper and lower extremities [3,4]. Some of such patients are referring to nephrologists to evaluate their secondary hypertension [5,6].

We recently encountered four patients who were referred to excluding the renovascular hypertension. Parvus-tardus waveform was demonstrated in Doppler Ultrasonography (DU) study without evidence of Renal Artery Stenosis (RAS). So in such cases, the presence of a parvus-tardus pattern in both renal arteries on DU should suggest the diagnosis of AC or aortic valve stenosis, that AC diagnosis was made at last by further evaluation for our cases.

## Methods

During April 2006 – September 2007, four consecutive patients were recruited for diagnostic work-up of secondary arterial hypertension by renal artery DU. Study participants met the following inclusion criteria: 1) arterial hypertension at age <30 years; 2) referred for renal Doppler to rule out secondary hypertension. DU was performed by Hitachi model EUB 525 (Hitachi Medical Corp, Tokyo, Japan) using convex probe 3.5-5 MHz.

In one patient, a 10-year-old female, Computed Tomography (CT) angiography was performed on suspition to abdominal AC by multislice spiral CT (Siemens, 64 slice, Germany), that was applied automatically in base of children protocol in agreemnet with ALARA.

## Cases presentation

### Case 1

**Figure 1:**
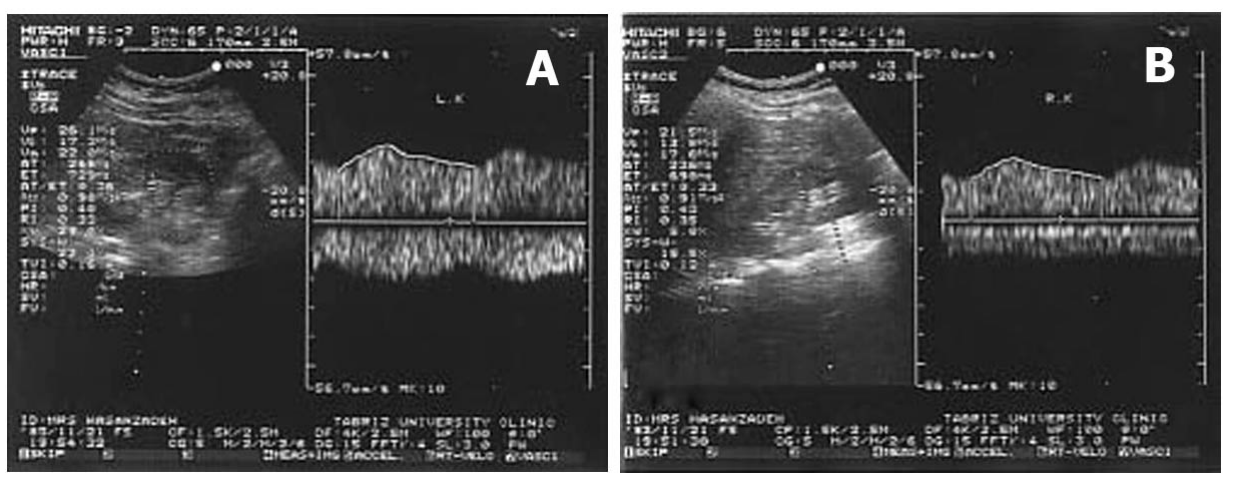
**(A) **Parvus-tardus pattern in left renal artery. **(B)** Similar parvus-tardus pattern is demonstrated in right renal artery.

**Figure 2:**
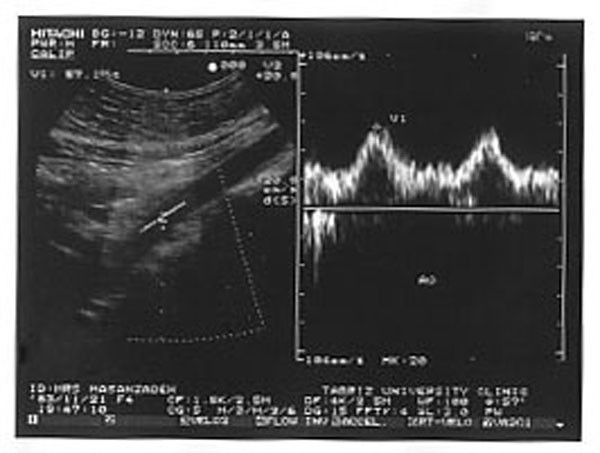
Extention of parvus-tardus waveform to abdominal aorta.

**Figure 3:**
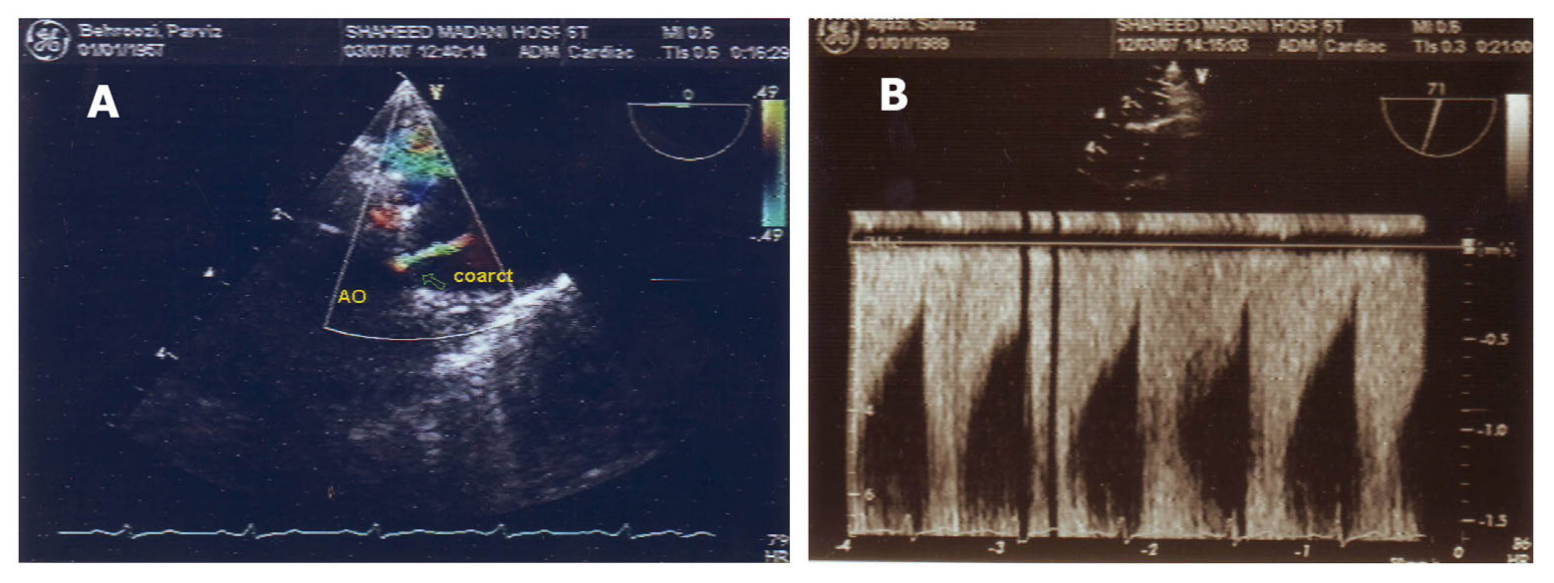
**(A)** Evaluation by TEE revealed aortic caorctation. **(B) **Recorded waveforms by cardiologist at post aorta coarctation locations.

A 21-year-old male with six-month history of tacking beta blocker. He was referred by a general practitioner for investigation of his secondary hypertension. This suspicion arises from negative family history and risk factor, and development of resistant hypertension. During physical examination, BP was 160/100 mmHg in his left brachial. According to these findings and the assumption of RAS, DU evaluation of renal arteries was recommended. During Doppler examination, parvus-tardus pattern was demonstrated in both renal arteries (Fig. 1A, B) and extention to aorta (Fig 2), but no evidance compatible with renal artries stenosis was detected. Trans-Esophageal Echocardiography (TEE) revealed a stenotic region about 2 cm length in the thoracic aorta distal to the origination of left subclavicular artery (Fig. 3A, B). After diagnosis, there was a 60 mmHg differences between upper (brachial) and lower (popliteal) extremities systolic BP. Finally, his BP normalized after successful surgery procedure.

### Case 2

A 27-year-old man was referred by nephrologists for evaluation of secondary hypertension. He did not report a previous history of abdominal surgery but the recent ultrasonographic study showed only one kidney in right hand side, so congenital agenesis of left kidney was suggested for him. Despite the parvus-tardus waveform pattern in intra-renal arteries through DU study, renal artery DU findings did not disclose any evidence of stenosis. This pattern continued along the renal artery to aorta which suggested an aortic obstruction. Consequently an aortic stenosis, distal to the origination of left subclavian artery, was reported using echocardiography and angiography. Physical examination also revealed 70 mmHg systolic BP difference between upper and lower limbs.

### Case 3

A 10-year-old female was referred by an urologist to work up her hypertension. Her small size right kidney was removed four months prior to perform DU on the assumption of hypertension. On the admission day, her brachial BP was 190/90 mmHg. In DU study, a parvus-tardus pattern was revealed in left kidney without any evidence of stenosis and which extended to aorta. During examination of abdominal aorta, stenotic region was detected just after emergence of celiac artery from aorta. According to this Doppler findings, for determination of involved region and confirmation of diagnosis, CT angiography was performed that demonstrated a 20.3 mm aortic stenosis zone that was started just after the emergence of celiac artery. Both left renal artery and inferior mesenteric artery originated from the stenotic region. However, the origin of the left renal artery had a 3.7 mm diameter without any stenosis through its length. After makeing diagnosis, a significant difference (80 mmHg) was found between brachial and popliteal systolic BP. Following surgical treatment, the stenotic region in aorta was removed and left renal artery was reinserted to aorto by dacron graft.

### Case 4

A 17-year-old female who was referred by a neurologist because of sever headache and hypertension (brachial BP: 180/110 mmHg) to follow for secondary hypertension. Bilateral parvus-tardus waveform was demonstrated by sonologist in renal arteries DU study. The same pattern was also revealed in DU study of abdominal aorta. Additional evaluation by TEE showed an estimated 2 cm length and 0.2 cm diameter AC located distal to the origin of left subclavicular artery. After definitive diagnosis, her popliteal BP was 100/70 mmHg. Following surgical intervention, BP returned to normal range.

## Discussion

For all cases, suspicion of aortic stenosis was made by DU rather than physical examination. Thereafter, we found that the hallmarks of significant AC were easily overlooked in all four cases by physicians. A comprehensive physical examination at the first visit, brachial systolic BP 10 mmHg greater than popliteal artery BP and radio-femoral pulse delay would have provided sufficient finding to raise the suspicion of AC, so that medical guidelines recommended a comprehensive medical history and physical examination. The results of this study are consistent with Cuspidi et al. study [7] that decision making following BP measurement limited to only one arm could lead to wrong diagnosis.

**Figure 4:**
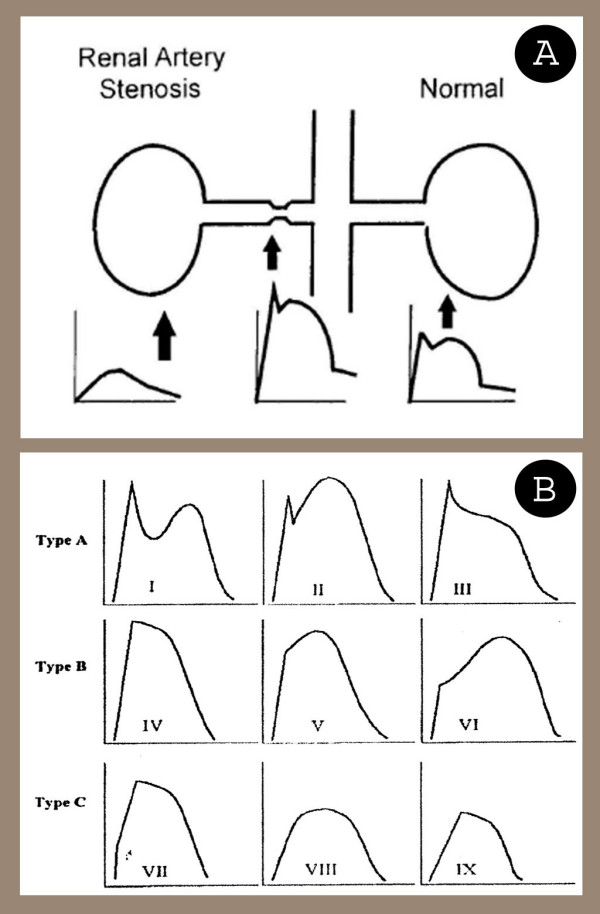
**(A) **Schematic view of different waveforms was showed according to stenosis location (From third edition of Dignostic Ultrasound by Rumak C. M, et al.). **(B) **Various types of Doppler waveforms. Type A and B are normal types, but type C patterns called parvus-tardus (From second edition of Dignostic Ultrasound by Rumak C. M, et al.).

The second important aspect is concerning the diagnostic value of renal DU in detecting AC. The majority of sonologists introduced the dampened waveform pattern known as parvus-tardus [6]. This pattern mostly detected through on indirect assessment of arterial region located after stenosis (Fig. 4A) [8]. Tardus means slow and late and parvus means small and little. Tardus refers to the facts that systolic acceleration of the waveform is slowed, with consequent increase in time to reach the systolic peak. Parvus refers to the fact that the systolic peak is of low height, indicating slowed velocity. Poststenotic systolic peak are rounded with lengthened systolic rise time (or slow systolic acceleration time – the time in seconds from the onset of the systolic to peak systole), slower than 0.07 s. the acceleration index (the slope of systolic upstroke) is decreased to lesser than 3 m/s^2 ^[9]. Normal and parvus-tardus waveforms were demonstrated in figure 4B. As shown, type A and B are normal patterns, whereas type C shows three schematic forms of parvus-tardus pattern.

Type C waves show a significant stenosis from aorta to renal segmental arteries. Only one side involvement reveals that side renal artery stenosis. In case of parvus-tardus pattern in both side, abdominal aorta waveforms need to assessed. Absence of this pattern at abdominal aorta in presence of renal arteries parvus-tardus waveform demonstrates bilateral renal arteries stenosis, however, extension of this pattern to aorta can also be seen in cases of aorta stenosis, left-sided heart failure and left ventricular outflow obstruction such as aortic valve stenosis [10]. Therefore, although it would be really convenient to simply evaluate the flow changes in the kidneys and thereby making diagnosis of renal artery stenosis without the arduous task of finding and directly assessment of the renal arteries, but parvus-tardus presence in renal artery may be suggests the other deferential diagnosis, as described above. Thus, we need to determine this pattern in both sides and confirm by direct assessment [11].

For cases reported here, spectral waveform analysis of the renal arteries and abdominal aorta revealed decreased flow, consistent with a parvus-tardus pattern. The abdominal aortic waveform helped us to rule out bilateral renal artery stenosis and suggested a proximal stenosis in the aorta.

These DU findings do not define the location of stenosis other than proximal to the evaluated artery but it could be due to a stenosis more proximal to the renal arteries, as shown in our four cases. This should be considered when there is a bilateral intrarenal parvus-tardus arterial wave pattern, particularly when this pattern extends through the renal arteries and aorta [5]. These latter two components could help diagnosis, particularly in people with single kidney. The presence of a bilateral parvus-tardus waveform pattern or unilateral in a single kidney patient always should led us to think more proximal, so that, valvular stenosis can role out and AC diagnosed definitely by echocardiography or CT angiography.

## Conclusion

In our patients, diagnosis of aortic forestation was made using ultrasonography rather than physical examination. Described diagnostic process is reflecting a common negligence of different physicians involving in management of hypertension, but diagnosis of AC by DU is vigilance of sonologist. So in patients who referred for Doppler evalaution on suspision of renovascular hypretension, in case of presence of bilateral parvus-tardus pattern without direct signs of stenosis, AC should be considered as differential diagnosis.

## Abbreviations

AC – Aortic coarctation

ALARA – As low as reasonably achievable

BP – Blood pressure

CT – Computed tomography

DU – Doppler ultrasonography

RAS – Renal artery stenosis

TEE – Trans-esophageal echocardiography

## Competing interests

The author(s) declare that they have no competing interests.

## Authors' contributions

MKT conceived the paper, and carried out the Doppler ultrasound.

NN followed up the patients, drafted and revised the manuscript.

MRA is nephrologist who one of patients was under care of him.

JE is nephrologist who two patients were under care of him.

HN is nephrologist who one of patients was under care of him.

FGS performed Doppler ultrasonographic evaluation.

MT performed trans-oesophageal echocardiography.

All authors read and approved the final manuscript.
